# Identification of differentially expressed genes and splicing events in early-onset colorectal cancer

**DOI:** 10.3389/fonc.2024.1365762

**Published:** 2024-04-11

**Authors:** Olivia M. Marx, Marc M. Mankarious, Walter A. Koltun, Gregory S. Yochum

**Affiliations:** ^1^ Koltun and Yochum Laboratory, Department of Surgery, Division of Colon & Rectal Surgery, Pennsylvania State University College of Medicine, Hershey, PA, United States; ^2^ Department of Biochemistry & Molecular Biology, Pennsylvania State University College of Medicine, Hershey, PA, United States

**Keywords:** early-onset, colorectal cancer, late-onset, splicing, transcriptome

## Abstract

**Background:**

The incidence of colorectal cancer (CRC) has been steadily increasing in younger individuals over the past several decades for reasons that are incompletely defined. Identifying differences in gene expression profiles, or transcriptomes, in early-onset colorectal cancer (EOCRC, < 50 years old) patients versus later-onset colorectal cancer (LOCRC, > 50 years old) patients is one approach to understanding molecular and genetic features that distinguish EOCRC.

**Methods:**

We performed RNA-sequencing (RNA-seq) to characterize the transcriptomes of patient-matched tumors and adjacent, uninvolved (normal) colonic segments from EOCRC (n=21) and LOCRC (n=22) patients. The EOCRC and LOCRC cohorts were matched for demographic and clinical characteristics. We used The Cancer Genome Atlas Colon Adenocarcinoma (TCGA-COAD) database for validation. We used a series of computational and bioinformatic tools to identify EOCRC-specific differentially expressed genes, molecular pathways, predicted cell populations, differential gene splicing events, and predicted neoantigens.

**Results:**

We identified an eight-gene signature in EOCRC comprised of *ALDOB*, *FBXL16*, *IL1RN*, *MSLN*, *RAC3*, *SLC38A11*, *WBSCR27* and *WNT11*, from which we developed a score predictive of overall CRC patient survival. On the entire set of genes identified in normal tissues and tumors, cell type deconvolution analysis predicted a differential abundance of immune and non-immune populations in EOCRC versus LOCRC. Gene set enrichment analysis identified increased expression of splicing machinery in EOCRC. We further found differences in alternative splicing (AS) events, including one within the long non-coding RNA, *HOTAIRM1*. Additional analysis of AS found seven events specific to EOCRC that encode potential neoantigens.

**Conclusion:**

Our transcriptome analyses identified genetic and molecular features specific to EOCRC which may inform future screening, development of prognostic indicators, and novel drug targets.

## 1 Introduction

The incidence of EOCRC in patients under the age of 50 is increasing world-wide (1, 2). Environmental factors, such as a Western diet and obesity, have been associated with CRC development at an earlier age (3, 4). As many CRC screening programs do not begin until the age of 45-50 years old (5, 6), there is often a delay in the diagnosis of these patients (7), and cancer is not detected until a later stage when it is associated with higher mortality (8). Interestingly, this stage difference cannot be fully explained by the diagnosis delay, pointing to differences in underlying pathophysiology (8). Treatment options largely remain the same for CRC patients of different ages (9), though EOCRC and LOCRC are often molecularly and physiologically distinct. In comparison to LOCRC, EOCRC tumors are more likely to have signet ring morphology (7, 10), are more often located in the distal colon or rectum (7), and have different mutational frequencies, including a reduced incidence of tumors with *APC* mutations (11, 12). Further understanding these molecular differences between EOCRC and LOCRC could improve early detection and treatment for the growing population of EOCRC patients.

While mutational comparisons between young and old CRC patients have been performed previously (11, 12), only very recent studies have examined the transcriptional profiles of EOCRC compared with LOCRC (13–16). These studies identified a variety of differences between EOCRC and LOCRC, including differences in immune signature (14, 15) and predicted immunotherapy response (13). Other studies have found no such differences (17) and instead found differences in DNA damage response (17) or oxidative stress response (18). Some studies have implicated specific genes involved in EOCRC such as *ALDH1A1* (19, 20), *PEG10* (21), or *ANPEP* (22). Many previous EOCRC transcriptomic studies examine differences between EOCRC and LOCRC tumor samples (19, 21–23), leaving it unclear whether results are important for cancer progression or artifacts of aging tissues. Other studies may have matched control samples but do not control for patient characteristics between EOCRCs and LOCRCs (17), allowing for differences between patient populations, such as stage, gender, tumor location, and histology, to drive EOCRC versus LOCRC differences (13). The variety of genes and gene signatures identified in previous studies may be reflective of different study populations and designs, highlighting the need for a well-controlled study of the EOCRC transcriptome.

Another limitation to previous EOCRC studies is the limited analysis of post-transcriptional modifications (24). Modifications such as alternative splicing (AS) are important contributors to CRC (25, 26) and are known to change in response to age, environmental stimuli (27, 28), and the microbiome (29). Although tumor-specific splicing factors and alternatively spliced transcripts show promise as therapeutic targets and biomarkers for CRC (30, 31), AS has not been studied in EOCRC (24). To overcome these limitations, we propose a strategy to examine differential gene expression and splicing in EOCRC and LOCRC tumors compared with matched adjacent normal tissues. Other than differences in age at diagnosis, clinical and demographic characteristics are also matched between the EOCRC and LOCRC patient cohorts. This careful study design allows us to identify transcripts that are differentially regulated in EOCRC that may act as therapeutic targets and provide insight into EOCRC pathogenesis.

RNA-seq is a robust tool to understand not only gene expression but can also be used to predict cell type composition and alternatively spliced genes. In this study, we selected a LOCRC cohort that matched our previously published EOCRC cohort (16) and compared transcriptomes and splicing via bulk RNA seq of tumors and adjacent full-thickness normal colonic tissues. We identified an eight-gene signature in EOCRC. We found several immune-related genes were differentially expressed in EOCRC compared with LOCRC and confirmed their correlation with age in the TCGA COAD dataset. Gene set enrichment analysis identified enrichment of spliceosome factors in EOCRC samples, and we further identified differential AS events encoding tumor-specific neoantigens, some of which were predicted to strongly bind the major histocompatibility complex (MHC). Together, our analysis of EOCRC versus LOCRC transcriptomes could help inform future biomarkers and therapeutic targets for the growing population of EOCRC patients.

## 2 Materials and methods

### 2.1 Specimen collection

Specimens were collected as previously described (16). Briefly, patients gave informed consent prior to undergoing surgery. Surgically resected tumors and full-thickness adjacent and uninvolved colonic tissues (hereafter referred to as normal) were stored in RNAlater and saved in our Carlino Family Inflammatory Bowel and Colorectal Disease Biobank within the Department of Surgery at the Pennsylvania State University College of Medicine. Full-thickness tissues from the rectum, sigmoid colon, ascending colon, descending colon, and cecum were collected, and only the raised portion of the tumor was used for sequencing to minimize contamination from normal cells. Microsatellite stability was determined based on current clinical guidelines and corresponding clinical information was collected. The Pennsylvania State University College of Medicine Institutional Review Board approved this study (IRB Protocol No. STUDY00021556).

### 2.2 RNA-sequencing and alignment

EOCRC tissue RNAs were sequenced in a previous study (16) and an additional 22 pairs of LOCRC patient tumors and adjacent normal tissues were likewise sequenced in the current study. Full-thickness tissue from surgically re-sectioned samples from throughout the colon and rectum were collected, flash frozen and stored in RNAlater. Total RNA extraction was performed as follows: tissues were homogenized with TRIzol, vortexed with chloroform, and centrifuged at 12,000 x g for 15 mins at 4°C. The aqueous layers were mixed with 70% ethanol and RNAs were purified further using Qiagen RNeasy Mini or Midi kits. RNA integrity numbers were assessed and RNA-seq libraries were prepared in the Penn State College of Medicine Genome Sciences core (RRID : SCR_021123) using the Illumina Stranded mRNA Prep, Ligation kit according to the manufacturer’s instructions. The libraries were pooled and sequenced on Illumina NovaSeq 6000, to obtain an average of 30 million, paired-end 100 bp reads. FASTQC was used to examine data quality, and all Phred scores were over 30 with no GC bias. Reads were aligned to the ensemble hg38 reference genome using STAR version 2.7.3 with default parameters (32). **Figure S1** summarizes quality control measures for EOCRC and LOCRC cohorts. HTSeq was used to count aligned reads (33). The gene expression omnibus (GEO) repository GSE196006 contains the sequencing data from the EOCRC cohort (16), and GSE251845 contains the sequencing data from the LOCRC cohort generated in this study.

### 2.3 Differential expression analysis

Differentially expressed genes were calculated with DESeq2 (34) separately for both EOCRC and LOCRC tumors versus normal samples. Normalization of EOCRC and LOCRC tumor and normal samples was performed through the variance stabilizing transformation (vst) function of the DESeq2 package. Log_2_ fold change was calculated for each patient by subtracting the log_2_ normalized adjacent (normal) values from log_2_ normalized tumor values for each gene. Gene ontology (GO) analysis was performed as previously described (16) with the clusterprofiler and Enrichplot packages in R (35). Gene set enrichment analysis (GSEA) was run using GSEA version 4.1.0 with default parameters (36). Heatmaps were created using the pheatmap function in R using unsupervised hierarchical clustering and scaling by row unless otherwise noted. Principal component analysis was run with the DESeq2 plotPCA command for the top 5000 most variable genes using gene expression data and the prcomp command in R stats package was used for PCA of cell deconvolution and PCA of percents spliced in results (37).

We considered genes significantly deregulated in tumors if the adjusted *P*-value, *P-*adj< 0.05, log_2_ fold change (LFC) > 1 (upregulated) or LFC< -1 (downregulated), and the mean reads were greater than 50. We considered LOCRC genes not significantly differentially expressed when *P-*adj > 0.2, LFC< 0.7, and mean reads > 50. When comparing differentially expressed genes in EOCRC and LOCRC, we also included genes with a LFC difference in EOCRC versus LOCRC greater than 1.5, where either the absolute value of LFC in EOCRC was greater than in LOCRC, or EOCRC was upregulated while LOCRC was downregulated or vice versa. This ensures the differential gene expression was more robust in EOCRC compared with LOCRC.

### 2.4 TCGA COAD analysis

Transcriptome data were downloaded from TCGA COAD study using the TCGAbiolinks (38) package as previously described (16) (TCGA Research Network. Available online: https://www.cancer.gov/tcga). As age at diagnosis is noted in days in TCGA COAD data obtained from the TCGAbiolinks download, we considered an age at diagnosis at or under 18,250 days (approximately 50 years) as EOCRC, and above 18,250 days as LOCRC. We analyzed primary tumors and solid normal tissues with age data available and normalized the data with the DESeq2 vst function (34). Kaplan-Meier (K-M) curves were created using TCGA COAD primary tumor expression data downloaded from the TCGAbiolinks package and survival information downloaded from the UCSC Xena platform (39). The survival package (40) (version 3.5-5) was used to generate a predictive score for COAD survival based on tumor expression of eight genes unique to EOCRC using a Cox proportional hazard model. A risk score was generated as below, by multiplying the coefficients (C) from the Cox regression by the expression (E) of each gene and taking the sum for each tumor sample.


$$risk\;score=C_{1}E_{1}+C_{2}E_{2}+C_{3}E_{3}+C_{4}E_{4}+C_{5}E_{5}+C_{6}E_{6}+C_{7}E_{7}+C_{8}E_{8}$$

Samples were separated into high and low groups based on median risk score, where patients with scores greater than or equal to the median score were in the high-risk group, and patients with scores less than the median were in the low-risk group. K-M curves for individual gene expression were similarly broken into high and low groups based on median tumor expression of each gene. The K-M curve *p*-values were calculated using the log-rank test.

### 2.5 Cell type deconvolution

Cell-type deconvolution was performed with XCell (41), which predicts the proportions of 64 cell types in bulk RNA-seq samples based on gene expression profiles characteristic of each cell. A paired Wilcoxon test was used to compare cell scores between tumor and matched normal samples. An unpaired Wilcoxon test was used to compare cell scores between EOCRC and LOCRC groups and unmatched tumor samples from the TCGA COAD dataset. To calculate scores, we divided the sum of the lymphoid, myeloid, or other cell types by the total sum of the cell scores for each patient, giving the average proportion of cell scores for each cell grouping. Boxplots were made using ggplot2 in R (42).

### 2.6 Consensus molecular subtyping

Consensus molecular subtyping (CMS) was performed on tumor data using the R package CMScaller (43). Gene names were converted from Ensembl ID to Entrez ID numbers using the bitr command from the clusterProfiler package (35). CMScaller was run using default parameters, with RNAseq = TRUE. Fisher’s exact test was used to compare CMS between different patient groupings, either by age (EOCRC versus LOCRC), or by expression of *ALDOB*, *WNT11*, *MSLN*, *RAC3*, *IL1RN*, *FBXL16*, *WBSCR27*, and *SLC38A11* above (high) or below (low) the median expression.

### 2.7 Splicing analysis

The rMATS -turbo (44) tool was run for EOCRC and LOCRC tumor versus normal samples. Default parameters were used in conjunction with STAR (32) (2.7.3a), miniconda (version 3), and python 3.8.13. Results for splice events included junction and exon counts. The hg38 reference genome was used, and the variable-read-length parameter was included, as reads were not trimmed prior to analysis. rMATS2Sashimiplot was used to generate sashimi plots of combined tumor and normal. bam files, generated with samtools merge on all. bam files, version 1.17. To examine significantly changed splice events, we filtered events for false discovery rate (FDR)< 0.05 and change in percent spliced in (PSI) > 0.1 in tumors versus normal samples. To ensure adequate read coverage, we further filtered for events with an average coverage of over 20 reads in tumor samples. As an independent validation, we repeated the splicing analysis with Whippet (45), a transcript-level approach to splicing analysis. Quantified Whippet results were subsequently filtered for splice events with delta PSI > 0.1 in tumors versus normal samples and calculated probability > 0.7. We then compared the starting or ending coordinates of the splice events identified with rMATS and Whippet analyses, keeping only splice events that were significant in both analyses. The rMATS value denotes exon location with a zero-based method (for example, denoting the last base pair of an intron before the actual exon starts) while Whippet provides one-based sites (denoting the location of the exon start), so the number one was added to the rMATS start coordinate to match the Whippet coordinate. The alternative 3’ splice types were described differently between the two programs, and the ending coordinate from Whippet was matched with the ending coordinate from rMATS. Notably, over 75% of rMATS reads for each splicing type (skipped exon, alternative 3’ splice site, alternative 5’ splice site, mutually exclusive exons, and retained introns) matched to a corresponding read from Whippet, suggesting minimal loss of information due to different naming conventions between the two programs. EOCRC splice events were considered uniquely differentially regulated if they were significant in both rMATS and Whippet analyses for EOCRC tumor versus normal and not significant in both rMATS and Whippet analyses for LOCRC tumor versus normal.

### 2.8 Neoantigen prediction

We used our list of 82 significant splice events in EOCRC tumor versus normal samples to predict tumor-specific neoantigens. We created a bed12 file with the locations of the alternatively spliced regions that were significantly more highly expressed in EOCRC tumors versus normal. We also included 27 flanking nucleotides to fit with downstream analysis. We used bedtools getfasta (46) command to obtain a corresponding fasta file with -s and -split arguments to ensure results reflected the proper strand and that results did not include intronic sequences not involved in splice sites, respectively. These fasta sequences were translated into amino acid sequences using EMBOSS transeq over the three forward reading frames (47). We removed resulting amino acid sequences with stop codons and submitted the remaining peptides to netMHCpan 4.0 (48) to predict binding to four common HLA subtypes (HLA-A*01:01, HLA-A*02:01, HLA-A*03:01, and HLA-A*24:02) using a 9mer peptide length. Amino acid sequences predicted to strongly bind any HLA type tested (top 0.5% of results) were considered potential neoantigen targets.

### 2.9 Statistics

Statistical tests were performed in R version v4.1.1 (37). Paired Wilcoxon tests were used to compare gene expression in matched tumor and adjacent uninvolved colonic segments. Unpaired Wilcoxon tests were used to compare gene expression in EOCRC and LOCRC groups. Significance of *p<* 0.05 was used for two-tailed statistical tests unless otherwise described. Venn diagrams of overlapping gene lists were made using the ggvenn package in R. Chi-square tests were used to determine clinical correlations with EOCRC versus LOCRC and within the eight-gene signature. Pearson correlation was used to assess the linear correlation of gene expression with age in days from tumor samples from the TCGA COAD dataset. Fisher’s exact test was used to compare Consensus Molecular Subtypes between patient groups.

## 3 Results

We designed this study to specifically identify age-related transcriptomic differences between tumors found in EOCRC and LOCRC patients. We expanded upon our previously published analysis of 21 patient matched EOCRC tumors and adjacent normal colonic/rectal segments (16). Here, we identified a cohort of 22 LOCRC patients that matched the EOCRC patients to compare the transcriptomes of CRC patients who differ primarily in age. We ensured that no significant differences in demographic information or clinical characteristics were present in our cohorts, including gender, ethnicity, body mass index, smoking history, tumor stage, microsatellite instability status, and tumor location (**Table 1**). Our approach therefore accounts for potential confounding effects due to clinical variability.

**Table 1:** Patient and Disease Characteristics.

	Early	Late	p
N	21	22	
Age at Operation (median [IQR])	45.50 [39.80, 47.80]	68.21 [55.65, 80.75]	<0.001
Male Gender (%)	12 (57.1)	15 (68.2)	0.665
Race (%)			0.38
Asian	1 (4.8)	0 (0.0)	
Black	1 (4.8)	0 (0.0)	
Other	0 (0.0)	1 (4.5)	
White	19 (90.5)	21 (95.5)	
BMI (median [IQR])	31.70 [25.70, 34.70]	29.00 [26.05, 32.75]	0.67
BMI Classification (%)			0.275
Normal	5 (23.8)	3 (13.6)	
Obese	12 (57.1)	10 (45.5)	
Overweight	4 (19.0)	9 (40.9)	
Tobacco Use (%)			0.251
Current User	2 (9.5)	5 (23.8)	
Ex User	6 (28.6)	8 (38.1)	
Never Used	13 (61.9)	8 (38.1)	
Right-sided Tumor (%)	6 (28.6)	4 (18.2)	0.656
Mucinous features (%)	3 (14.3)	3 (13.6)	1
Microsatellite Instability Present (%)	3 (14.3)	2 (9.1)	0.956
Grade (%)			0.197
Moderate	11 (52.4)	8 (36.4)	
Poor	3 (14.3)	1 (4.5)	
Well	7 (33.3)	13 (59.1)	
AJCC Stage At Presentation (%)			0.2
1	6 (28.6)	8 (36.4)	
2	3 (14.3)	7 (31.8)	
3	10 (47.6)	4 (18.2)	
4	2 (9.5)	3 (13.6)	
T-stage (%)			0.151
1	3 (15.0)	2 (9.1)	
2	4 (20.0)	6 (27.3)	
3	6 (30.0)	12 (54.5)	
4	7 (35.0)	2 (9.1)	
N-stage (%)			0.306
0	9 (45.0)	15 (68.2)	
1	7 (35.0)	4 (18.2)	
2	4 (20.0)	3 (13.6)	
M-stage (%)			1
0	18 (90.0)	19 (86.4)	
1	2 (10.0)	3 (13.6)	
Lymphovascular Invasion Present (%)	6 (30.0)	3 (13.6)	0.219
Perineural Invasion Present (%)	6 (30.0)	1 (4.5)	0.072
Recurrence (%)	6 (28.6)	2 (9.1)	0.212
Time to Recurrence (Days) (median [IQR])**	321.00 [177, 376.5]	282.00 [242, 322]	0.739

IQR, interquartile range; BMI, Body Mass Index; AJCC, American Joint Committee on Cancer. Missing values omitted from calculations. **Calculated only for patients who had a recurrence

### 3.1 Identifying a differentially expressed gene signature in EOCRC

**Figure 1:**
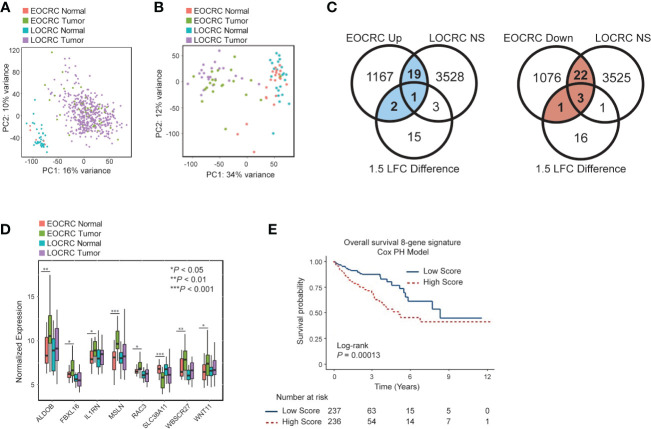
Identifying a differentially expressed gene signature in EOCRC **(A)** Principal component analysis (PCA) for EOCRC and LOCRC tumor and normal samples from TCGA COAD dataset. **(B)** PCA of EOCRC tumors (n=21 green) and normals (n=21 coral) and LOCRC tumors (n=22 purple) and normals (n=22 blue) top 5000 most variably expressed genes within our cohort. **(C)** Venn diagrams showing identification of uniquely differentially expressed genes in EOCRC vs LOCRC (left: upregulated, uniquely upregulated in blue; right: downregulated, uniquely downregulated numbers in red). Significance cutoffs were as follows: NS (not significant) LOCRC: adjusted *p*-value > 0.2, LFC< 0.7, mean counts > 50; EOCRC Up: adjusted *p-*value< 0.05, LFC > 1, mean counts > 50; EOCRC Down: *p*-value< 0.05, LFC< -1, mean counts > 50; 1.5 LFC difference is between EOCRC and LOCRC, where the absolute value of EOCRC is greater than LOCRC or EOCRC differential expression is significantly positive and LOCRC is significantly negatively regulated, or vice versa. **(D)** Boxplot of eight genes differentially expressed in EOCRC and unchanged in LOCRC by paired Wilcoxon test *P< 0.05; **P< 0.01, ***P< 0.001 **(E)** Kaplan-Meier curve showing the association of the eight-gene signature survival score with overall survival.

We began by comparing the transcriptomes of EOCRC and LOCRC samples through principal component analysis (PCA) to determine if normal tissues and tumors from different age groups formed distinct clusters. We performed PCA on the normalized gene expression within TCGA COAD (**Figure 1A**) and our dataset (**Figure 1B**). Both analyses showed separate clustering of normal and tumor samples but no clustering of young and old samples.

We next aimed to identify specific differentially expressed genes in EOCRC. Genes that were significantly deregulated in EOCRC and either not significant in LOCRC or had a 1.5 LFC difference in EOCRC versus LOCRC (n = 48; 22 upregulated and 26 downregulated) were considered specific to EOCRC (**Figure 1C**; **Supplementary Table S1**). To further narrow down this list, we performed a paired Wilcoxon test to compare tumors and adjacent normal tissues in each cohort and identified a list of eight genes differentially expressed in EOCRC (**Figure 1D**, Wilcoxon *P<* 0.05 in EOCRC and Wilcoxon *P* > 0.25 in LOCRC, or *P<* 0.05 in both EOCRC and LOCRC and the LFC were changed in opposite directions or had a greater than 1.5 LFC difference). Most of these eight genes have known roles in cancer, but some have not been studied in the context of CRC (**Supplementary Table S2**). Next, we performed chi-square tests to determine if any of these eight genes were associated with clinical characteristics such as BMI, CRC family history, gender, smoking, and metastasis. Significant results (*P<* 0.05) included associations with *WNT11* and *FBXL16* and body mass index (BMI), *MSLN* and mucinous adenocarcinoma, and *SLC38A11* and metastasis (**Table 2**). To further examine the importance of the eight-gene signature in CRC, we constructed a risk score based on a Cox proportional hazards model for the tumor expression of the eight genes and overall survival from the TCGA COAD dataset (**Figure 1E**). This model showed significant differences in survival between those with high and low scores (separated by median). Kaplan-Meier curves for each of the eight genes individually found that only *WNT11* and *WBSCR27* had significant relationships between expression and overall survival (**Supplementary Figure S2**). Further analysis shows that separating patients by median score acts as an independent prognostic indicator for colorectal cancer (**Supplementary Figure S3A**, **Supplementary Table S3**).

**Table 2:** Significant clinical correlates for eight genes unique to EOCRC.

Gene	Clinical Feature	High n (%)	Low n (%)	P-value
*FBXL16*	Obese	14/21 (66.7)	7/22 (31.8)	0.0477
*WNT11*	Obese	14/21 (66.7)	7/22 (31.8)	0.0477
*SLC38A11*	Metastasis present	5/20 (25.0)	0/22 (0.0)	0.0432
*SLC38A11*	Ever Smoked	14/21 (66.7)	6/21 (28.6)	0.0306
*MSLN*	Obese	14/21 (66.7)	7/22 (31.8)	0.0477
*MSLN*	Mucinous	6/21 (28.6)	0/22 (0.0)	0.0237

Chi-square test was used to determine significant association with clinical features. The combined cohort of EOCRC and LOCRC patients were split into high/low groups based on differential expression of each gene greater than the median (high) or less than or equal to the median (low). The numbers represent the number of patients positive for the clinical feature out of the number of patients with high or low expression of that gene with available data for that clinical feature.

We next validated our gene signature using the TCGA COAD dataset. As this dataset has limited normal samples from young patients, we used a Pearson correlation to identify genes correlating with age in the TCGA COAD dataset. Of the 2291 genes significantly differentially expressed in our EOCRC samples, 268 were significantly correlated with age (Pearson, *P<no><</no>* 0.05). Twelve of these genes overlapped with our 48 gene-signature, and this overlap was significant (Fisher test *P* = 0.0097, odds ratio 2.585 (95% confidence interval 1.208, 5.165), (**Supplementry Figure S3B**). One of these twelve genes was also present in our identified 8-gene signature, *FBXL16* (**Supplementry Figure S3C**). Therefore, the TCGA COAD dataset supports that the genes we identified have age-specific expression.

Colorectal cancer consensus molecular subtypes (CMS) provide a useful way to stratify CRC tumors. Here, we used CMSCaller to predict CMS based on transcriptomic data of our and TCGA COAD datasets. We found no significant difference in CMS subtypes between EOCRC and LOCRCs in our data (**Supplementry Figure S4A**) or TCGA COAD data (**Supplementry Figure S4B**), however, we did see similar trends in both datasets, with EOCRC having slightly more CMS4 and LOCRC having more CMS2. Hierarchical clustering based on tumor expression of our eight-gene signature did not appear to cluster by CMS (**Supplementry Figure S4C**). To further examine the relationship between CMS and our eight-gene signature, we stratified the data by median tumor expression of each gene and performed Fisher’s exact tests to determine whether high/low gene expression was associated with CMS (**Supplementary Table S4**). *IL1RN* expression was the only gene significantly associated with CMS in both datasets, and lower expression of *IL1RN* had a higher proportion of CMS2 tumors, which is enriched for Wnt/MYC activation, and higher *IL1RN* tumors had more CMS1, characteristic of immune activation (**Supplementry Figure S4D-E**).

### 3.2 Immune gene and cell type signatures distinguish EOCRC and LOCRC

**Figure 2:**
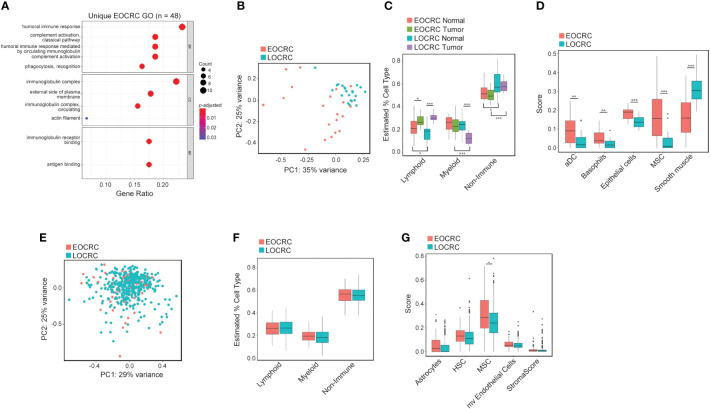
Immune gene and cell type signatures distinguish EOCRC and LOCRC. **(A)** Gene ontology of 48 genes uniquely differentially expressed in EOCRC and correlating with age in TCGA COAD dataset. **(B)** PCA of all cell types identified with XCell in tumors from EOCRC and LOCRC samples. **(C)** Boxplot of lymphoid, myeloid, and non-immune cell population comparisons in EOCRC and LOCRC tumors samples. **(D)** Comparison of top 5 differences in cell type score between EOCRC and LOCRC tumors (aDC,dendritic cells; MSC, mesenchymal stem cells). **(E)** PCA of all cell types identified with XCell in tumors from TCGA COAD EOCRC and LOCRC tumor samples. **(F)** Boxplot of lymphoid, myeloid, and non-immune cell population comparisons in EOCRC and LOCRC tumors from TCGA COAD. **(G)** Boxplot of the most differently expressed cell types in EOCRC and LOCRC tumors from the TCGA COAD dataset (HSC, hematopoietic stem cells; mv, microvascular). Wilcoxon test, *P< 0.05; **P< 0.01, ***P< 0.001.

To categorize the differentially expressed genes, we performed gene ontology (GO) analysis on the 48 genes unique to EOCRC and identified enrichment of immune-related terms (**Figure 2A**). This finding suggested that there may be differences in immune cell populations in EOCRC versus LOCRC samples. To investigate this possibility, we subjected the total set of normalized genes in all normal and tumor samples to cell type deconvolution analysis using the XCell program (41). PCA of predicted cell types clustered LOCRC and EOCRC tumors separately (**Figure 2B**). To understand the types of cells that differed between younger and older patients, we averaged the scores of lymphoid, myeloid, and other cell populations. In comparison to normal tissues, in tumors we found lymphoid populations were increased in both age groups, whereas myeloid cells were decreased in later-onset but not early-onset (**Figure 2C**). In both groups, non-immune cell types were unchanged in tumors versus normal tissues, but expression of these cell markers were significantly higher in later-onset samples (**Figure 2C**). Next, we identified the top five cell type differences in EOCRC and LOCRC tumors: dendritic cells (aDC), basophils, epithelial cells, mesenchymal stem cells (MSCs), and smooth muscle. Genes characterizing each of these cell types, with the exception of smooth muscle, were expressed at higher levels in early-onset versus later-onset tumors (**Figure 2D**).

To attempt to validate these findings, we performed the same XCell analysis on the TCGA COAD dataset. We found no differences in PCA clustering (**Figure 2E**), or lymphoid, myeloid, and non-immune populations in EOCRC versus LOCRC (**Figure 2F**). However, we did find enrichment of MSCs in early-onset versus later-onset tumors (**Figure 2G**). Therefore, our study design provides support for differences in immune and stromal cell populations in tumors and normal tissues of young and old CRC patients that were not seen in TCGA COAD patients.

### 3.3 Splicing is deregulated in EOCRC

**Figure 3:**
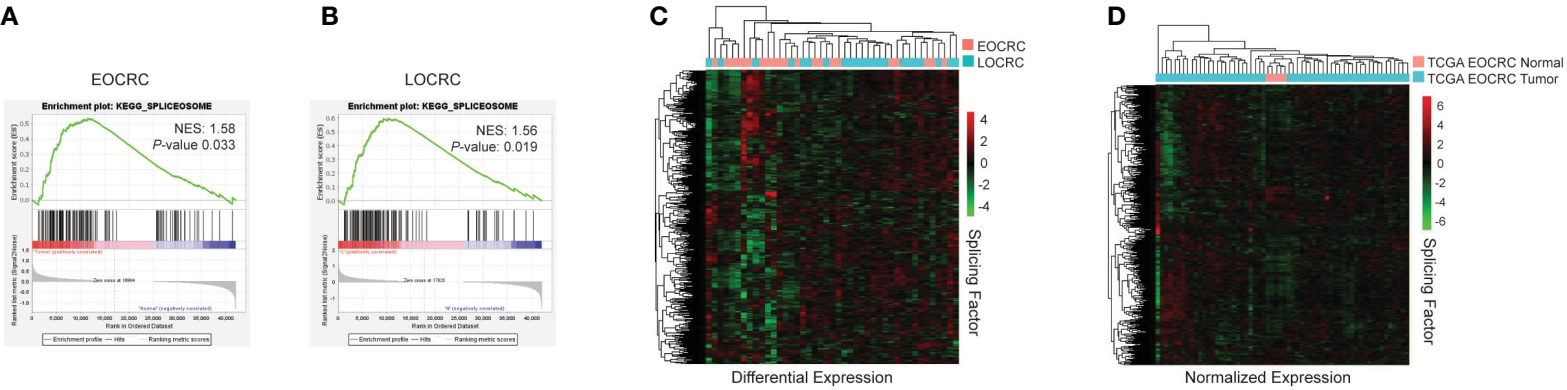
Splicing is deregulated in EOCRC. **(A)** KEGG analysis for spliceosome genes in EOCRC tumor vs normal, normalized enrichment score (NES) 1.58, nominal *P-*value 0.033. **(B)** KEGG analysis for spliceosome genes in LOCRC tumor vs normal, NES 1.56, *P-*value 0.019. **(C)** Heatmap of differential expression of GOBP_RNA_SPLICING genes in EOCRC and LOCRC patients with hierarchical clustering. **(D)** Heatmap of normalized expression of GOBP_RNA_SPLICING genes in normal and tumor samples from TCGA COAD EOCRC datasets with hierarchical clustering.

To further identify molecular and cellular pathways in our transcriptome dataset, we employed Gene Set Enrichment Analysis (GSEA) using the Kyoto Encyclopedia of Genes and Genomes (KEGG) (49) to examine our EOCRC tumors versus normal and LOCRC tumors versus normal samples. We found several common oncogenic pathways enriched in both EOCRC and LOCRC, including DNA replication, nucleotide excision repair, and spliceosome. However, we also found several unique terms within the EOCRC cohort, including positive enrichment of cell cycle and negative enrichment of the insulin signaling pathway and adipocytokine signaling (**Supplementary Table S5**). Given that splicing has not been investigated in EOCRC transcriptomes, we further evaluated genes comprising this category. Both EOCRC and LOCRC cohorts showed significant enrichment in the KEGG Spliceosome gene set (**Figure 3A, B**). To further examine RNA splicing factor expression, we next examined the differential expression of splicing factors between tumors and normal samples in EOCRC and LOCRC patients. We found that EOCRC and LOCRC patients clustered separately based on differential expression of RNA splicing genes from the GSEA GOBP_RNA_SPLICING dataset (36) (**Figure 3C**). Using the TCGA COAD dataset, we confirmed that splicing factor expression clusters tumor and normal samples separately from young patients (**Figure 3D**).

**Figure 4:**
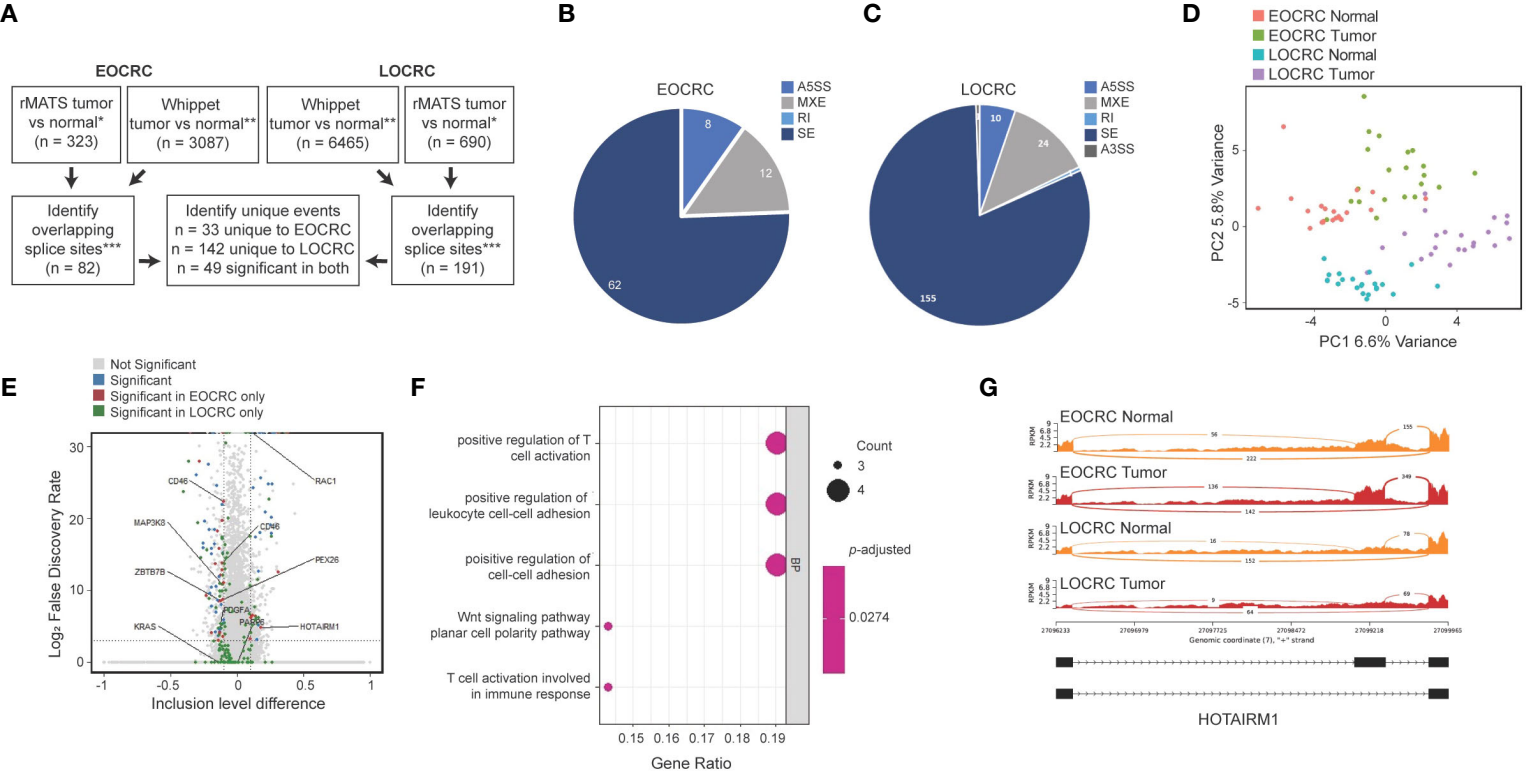
Alternatively spliced events in EOCRC differ from LOCRC. **(A)** Schematic of splice analysis strategy with splice events considered significant in rMATS tumor vs normal *rMATS average junction counts > 20, delta PSI > 0.1, FDR< 0.05; **Whippet delta PSI > 0.1, Probability > 0.7; ***merged by start location of splice event except A3SS which was merged by end location. **(B, C)** Pie chart of significant splice events identified in EOCRC **(B)** or LOCRC **(C)**, showing alternative 5’ splice sites (A5SS), mutually exclusive exons (MXE), retained intron (RI), skipped exon (SE), and alternative 3’ splice site (A3SS) events as defined by rMATS. **(D)** PCA of the top 5000 most variable splice events via rMATS analysis in EOCRC and LOCRC normal and tumor samples. **(E)** Volcano plot of rMATS results of EOCRC tumor versus normal differentially spliced events colored by those that were overall not significant (gray), significant in EOCRC only (red), significant in LOCRC only (green), or significant in both (blue). **(F)** Gene ontology of differentially spliced genes in EOCRC (n = 33). **(G)** Sashimi plot of combined EOCRC and LOCRC tumor and normal files showing *HOTAIRM1*.

To investigate AS events in younger and older CRC patients, we first identified splicing events that were more abundant in tumors versus normal samples. The results of two splicing analysis programs were merged to reduce false positive results (50). EOCRC and LOCRC sequencing data were processed using rMATS (44) and Whippet (45), and splice events were only considered significant if they were identified in both rMATS (average junction counts > 20, delta PSI > 0.1, false discovery rate (FDR)< 0.05) and Whippet (delta PSI > 0.1, Probability > 0.7) analyses (**Figure 4A**). We identified 82 significantly differentially spliced events in EOCRC tumors versus normal (**Supplementary Table S6**) and 191 in LOCRC, with 49 of these events present in both patient cohorts (**Figure 4A**). Differentially spliced events in EOCRC included 62 skipped exon events (SE), twelve mutually exclusive exon events (MXE), and eight alternative 5’ splice site events (A5SS) (**Figure 4B**). Differentially spliced events in LOCRC included 155 SE, 24 MXE, 10 A5SS, one alternative 3’ splice site (A3SS), and one retained intron (RI) splice event (**Figure 4C**). Principal component analysis found separation of EOCRC and LOCRC tumor and normal samples by PSI for the top 5000 most variable splice events (**Figure 4D**), further supporting age-related differences in CRC splicing.

We next constructed a volcano plot summarizing genes with differential splice events and highlight several genes of interest (**Figure 4E**). In support of our approach, we identified significantly deregulated splicing events in tumors versus normal samples, including pathogenic *KRAS* and *RAC1* variants, which are known to contribute to CRC (25). Among the 82 significant EOCRC AS events, we filtered out those that showed significant AS in LOCRC, leaving a list of 33 sites differentially spliced in EOCRC but not in LOCRC, including those in *ZBTB7B*, *PEX26*, *MAP3K8*, and *HOTAIRM1* (**Figure 4E**). Notably, no splice sites were significantly positively regulated in EOCRC and significantly negatively regulated in LOCRC or vice versa. These 33 genes with splicing specific to EOCRC were enriched for markers of T-cell regulation and cell-cell adhesion (**Figure 4F**). Of these genes, we highlight the long non-coding RNA (lncRNA) *HOTAIRM1* (**Figure 4G**), which has known roles in many cancers, including colorectal (51). In our samples, *HOTAIRM1* exhibited high exon inclusion in EOCRC tumors, lower exon inclusion in EOCRC normal samples, and very little exon inclusion in LOCRC tumors or normal samples. In total, these findings support age-specific differences in CRC splicing.

### 3.4 AS is a source of EOCRC-specific neoantigens

**Figure 5:**
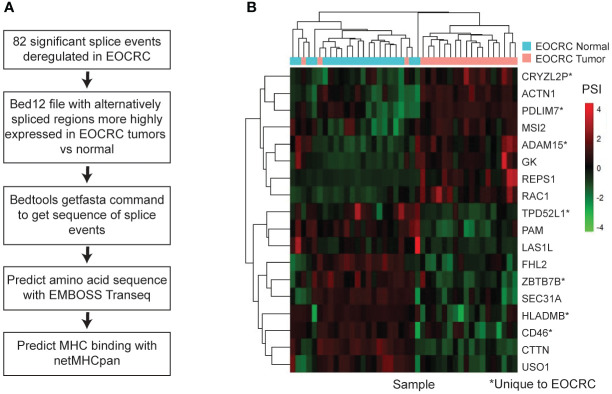
AS is a source of EOCRC-specific neoantigens. **(A)** Workflow to predict EOCRC-specific AS transcripts that may act as neoantigens. **(B)** Heatmap with PSI for tumor-enriched splice events predicted to bind the MHC I complex strongly in EOCRC normal and tumor samples.

To further explore the clinical relevance of AS in EOCRC, we assessed the potential of our identified splice events to produce neoantigens, or tumor-specific peptides that may get presented on the MHC complex and become a target for immunotherapy (52). We analyzed our list of 82 significantly differentially spliced events in EOCRC, identified the tumor-specific nucleotide sequence with bedtools, predicted the amino acid sequence with EMBOSS Transeq (47), and used netMHCpan (48) to identify the potential of EOCRC AS events to bind the MHC (**Figure 5A**). We identified 18 genes with tumor-specific splice events predicted to strongly bind to the MHC in at least one of four common HLA-A subtypes (**Figure 5B**). Of these, seven splice events were specific to EOCRC tumors versus normal. Together, these findings support diverse and clinically relevant differences between alternative splicing in EOCRC and LOCRC.

## 4 Discussion

In this study, we compared RNA sequencing of patient-matched tumors and adjacent normal tissues from EOCRC (n = 21) and LOCRC (n = 22) patients. Our usage of patient-matched tumor and normal samples gave us the unique ability to control for clinical and demographic features, which are known to contribute to EOCRC at an earlier age (3, 4, 53). We found several features unique to EOCRC, including differences in oncogene expression, predicted cell types, splicing factors and events, and predicted neoantigens.

Similar to previous studies, we did not identify large-scale transcriptomic differences between EOCRC and LOCRC (13). However, in this paper, we primarily aimed to identify genes that are differentially expressed in the tumors of EOCRC patients that are less prevalent in LOCRCs that may serve as biomarkers or therapeutic targets. In this study, we identified eight genes that were significantly differentially expressed in EOCRC but relatively unchanged in LOCRC. Of these, ALDOB, WNT11, MSLN, RAC3, and IL1RN have known roles in CRC (54–58), FBLX16 has known roles in other cancers (59, 60), and SLC38A11 and WBSCR27 are relatively uncharacterized (61–63). All of these genes may give insight into EOCRC pathogenesis and serve as prognostic markers or predict different drug responses compared to LOCRC. For example, MSLN is a target for CAR-T therapy that is currently being explored for CRC treatments (64). Furthermore, SLC38A11 may play a role in metabolism (61) and we identified a significant association between *SLC38A11* expression and metastasis in our cohort, suggesting it may be of clinical relevance, especially in EOCRC patients. Combined, these eight genes produced a risk score significantly associated with decreased overall survival, and our data suggest that this risk score may be an independent prognostic indicator of CRC. Other studies have generated EOCRC risk scores based on gene expression, and identified genes with EOCRC-specific expression however, their identified genes do not overlap with our gene list (13, 15, 23). This could be due to differences in sample sets and patient demographics, as well as study design. Furthermore, we did not identify differences in CMS between EOCRC and LOCRC samples in ours or the TCGA COAD datasets, although a previous study found higher CMS1 in patients under 40 years old (10). We did find that low *IL1RN* expressing tumors had an increased proportion of CMS2, characterized by canonical Wnt/β-catenin activation, and high *IL1RN* tumors had an increased proportion of CMS1, characterized by immune activation (65, 66). In addition to an eight-gene signature, we found a total of 48 genes uniquely differentially expressed in EOCRC versus LOCRC, 12 of which were correlated with age in the TCGA COAD tumor transcriptomic dataset.

Our 48 genes unique to EOCRC were enriched for immune-related GO terms, which is supported by previous transcriptomic profiling studies of EOCRC (14, 15). Furthermore, our cell type deconvolution identified age-related differences and is partially supported by recent transcriptomic studies that have examined immune cell signatures in EOCRC. One study by Lu et al. did not find a significant difference in immune cell deconvolution between EOCRC and LOCRC patients (13), while another study by Du et al. did find higher overall immune cell populations in EOCRC compared to LOCRC from a Chinese cohort (14). Immunosenescence, the process in which immune cells become dysfunctional with age (67), was suggested to contribute to the decrease in immune cells with aging (14). Here, we identified increased lymphoid populations in EOCRC and LOCRC tumors versus normal samples. We also found a decrease in myeloid cells in LOCRC tumors but no change in EOCRC tumors compared with normal samples. These findings further support that there are age and tumor-specific differences in CRC cellular profiles which should be further investigated, as immune infiltration is a critical prognostic indicator (68). These differences in predicted cell populations could support differences in immunotherapy response between EOCRC and LOCRC patients. Indeed, EOCRCs are known to have a higher mutational burden, and thus have been suggested to be more sensitive to immunotherapies (14). We could not confirm differences in myeloid, lymphoid, or other populations between EOCRC and LOCRC tumors from the TCGA COAD dataset, highlighting the novelty of our approach.

In addition to immune cell differences, we also found enrichment of dendritic cells, basophils, epithelial cells, and MSCs in EOCRC versus LOCRC samples. Several of these cell types have been shown to have prognostic value for CRC patients (69, 70). We identified MSC enrichment in EOCRC tumors compared with LOCRC in both ours and the TCGA COAD cohorts. Tumor-derived MSCs were shown to be recruited to the tumor microenvironment and promote CRC cell stemness, angiogenesis, and cytokine production (70). In contrast, other studies have found that bone-marrow-derived MSCs reduce cytokines and STAT3 activation, indicating a tumor supporessive role (71). These findings indicate that more research is needed to investigate the role of MSCs in colorectal carcinogenesis, which may be of potential importance to EOCRC patients.

We found enrichment of spliceosome-related genes in our EOCRC tumor versus normal samples. This was expected, as AS is known to play a role in CRC (25, 26). Our data found that differential expression of splicing factors clustered EOCRC and LOCRC patients separately. This clustering suggests different mechanisms of post-transcriptional regulation in EOCRC and LOCRC patients from our cohorts. Both aging and environmental factors could contribute to alterations in splice factor expression (27, 28). Despite this, AS has not previously been examined in EOCRC. Our sequencing contained approximately 30 million reads per sample without replicates, and thus, we took a conservative approach to detect limited AS events with high coverage in our dataset. Previous work has used PCA to examine AS-based differences in cancer subtypes (72). Indeed, our PCA showed separation of EOCRC and LOCRC tumor and normal samples based on PSI, with a 5.8-6.6% variance that may be suggestive of subtle splicing differences between EOCRC and LOCRCs. Even so, we identified previously annotated splicing events encoding known cancer related *RAC1* (73) variants in both the EOCRC and LOCRC cohorts. We also found a pathogenic *KRAS* variant that may be regulated by APC (25) unique to the LOCRC patients. As APC is a key regulator of CRC splicing (25), differences in *APC* mutational frequency between EOCRC and LOCRC (11, 12) may contribute to splicing differences between EOCRC and LOCRC.

We identified splice events unique to EOCRC that were enriched in T-cell activation, indicating potential for AS mediated differences in immune populations in EOCRC and LOCRC. Notably, we identified EOCRC tumor-specific splicing of *ZBTB7B* (*Th-POK*), a zinc finger transcription factor which controls T-cell differentiation into CD4+ or CD8+ T-cells (74), and *MAP3K8* (*TPL2*), a driver of oncogenic inflammation (75), though the biological function of these splice variants remains unknown. One splice variant unique to EOCRC with a potential known function was identified in the peroxidase biogenesis factor *PEX26*. We identified a decrease in exon 5 inclusion, consistent with the PEX26Δex5 isoform, which lacks a transmembrane domain and may be more involved in early peroxisomal biogenesis compared with the full length isoform (76). Splicing can be affected by RNA binding proteins or external factors, and thus, may be indicative of differences in tumor microenvironment or surrounding microbiome. Interestingly, we identified a splicing variant of the long non-coding RNA *HOTAIRM1*, which has been shown to be alternatively spliced in the presence of lipopolysaccharide, which is present in the cell walls of gram-negative bacteria (28). A few studies have examined *HOTAIRM1* in CRC and found it is downregulated and has potential as a serum biomarker (51, 77), though the role of the alternatively spliced transcript remains unknown.

More work is clearly needed to understand the biological causes and effects of many of the splice isoforms identified in this study. However, we were able to predict clinical relevance of peptides generated from AS events as a potential source of tumor-specific neoantigens. Here, we predicted the potential of EOCRC-specific AS transcripts to bind to the MHC complex and identified 18 potential strong binders, seven of which were specific to EOCRC compared with LOCRC. Splicing is an important source of future cancer vaccine targets (31), and our study suggests that these AS targets may be different for younger and older CRC patients. In addition, drugs altering global splicing changes have shown promise in CRC models (26), and our data suggests EOCRC patients may also benefit from these treatments.

Our study is not without limitations. The samples were collected at our institution between 2015-2021, so we have a limited sample size and incomplete survival information for each patient, making us unable to make judgments about prognostic impact of the gene expression, splice events, and predicted cell type profiles discussed here. Instead, we examined the prognostic impact of our gene signature using the TCGA COAD dataset, which is primarily composed of LOCRC samples, and may not necessarily reflect the genes’ prognostic impact for EOCRC samples. We also did not perform mutational profiling, and limited identification of specific EOCRC differentially expressed genes precluded us from performing network analysis that could connect samples to previously published genetic differences. In addition, we have limited data from non-white patients, making us unable to address the racial and ethnic disparities that exist in CRC and EOCRC research and clinical outcomes (78). Furthermore, we did not evaluate our targets in animal models of EOCRC to determine whether they are key determinants of colorectal carcinogenesis. Therefore, future work may focus on increased sample size, deeper sequencing to assess additional AS events, and long-term follow ups to increase the predictive power of the dataset. Furthermore, evaluation of the role of the genes *SLC38A11* and *WBSCR27*, as well as the functional roles of the alternatively spliced transcripts identified in this study in CRC could help clarify their roles in EOCRC progression.

In summary, this is the first transcriptomic study to our knowledge comparing the gene expression and splicing profiles of EOCRC and LOCRC patients with matched adjacent normal tissues and matched demographic information to ensure that the differences identified were tumor-specific and due to age. We identified uniquely differentially expressed genes, cell types, and splicing events that could help inform prognosis, treatment, and biomarkers for the growing population of EOCRC patients.

## Data availability statement

The original contributions presented in the study are publicly available. This data can be found here: GSE251845 and GSE196006.

## Ethics statement

The studies involving humans were approved by Pennsylvania State University Institutional Review Board. The studies were conducted in accordance with the local legislation and institutional requirements. The human samples used in this study were acquired from primarily isolated as part of your previous study for which ethical approval was obtained. Written informed consent for participation was not required from the participants or the participants’ legal guardians/next of kin in accordance with the national legislation and institutional requirements.

## Author contributions

OM: Writing – review & editing, Writing – original draft, Validation, Methodology, Investigation, Formal analysis, Data curation, Conceptualization. MM: Writing – review & editing, Investigation, Data curation. WK: Writing – review & editing, Supervision, Funding acquisition. GY: Writing – review & editing, Writing – original draft, Visualization, Supervision, Resources, Project administration, Investigation, Funding acquisition, Formal analysis, Conceptualization.
