# Supplementary material for: Identification of differentially expressed genes and splicing events in early-onset colorectal cancer
Source: Front Oncol. 2024 Apr 11;14:1365762. doi: 10.3389/fonc.2024.1365762 (PMC11047122; doi:10.3389/fonc.2024.1365762)
Supplement: Supplementary file 1 [file DataSheet_1.docx]

Supplementary Material


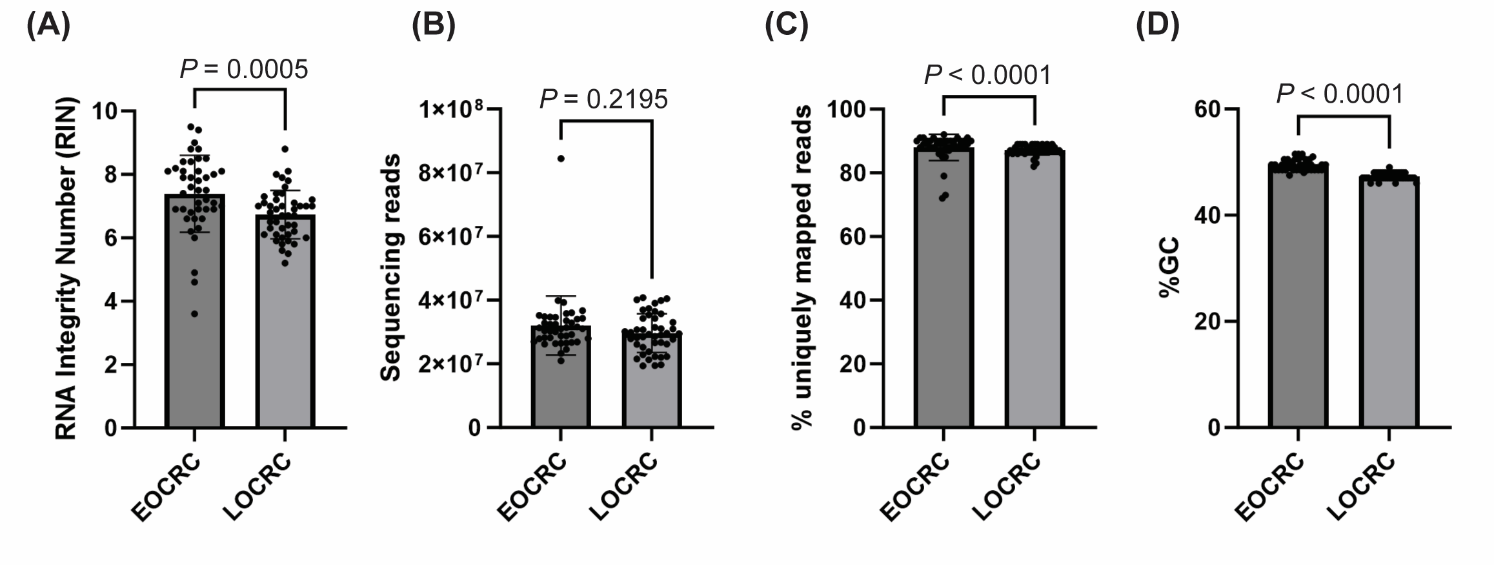


**Supplementary Figure 1:** RNA-sequencing quality control and mapping. (A) RNA integrity number for EOCRC (n = 42) and LOCRC (n = 44) samples. (B) The number of reads sequenced for each sample. (C) Percentage of uniquely mapped reads for each sample. (D) Percentage of GC content for each sample. Bars represent mean value, with points for each sample and error bars representing the standard deviation. *P*-values represent unpaired Wilcoxon test results, as all data was non-parametric by Shapiro-Wilks test.

**
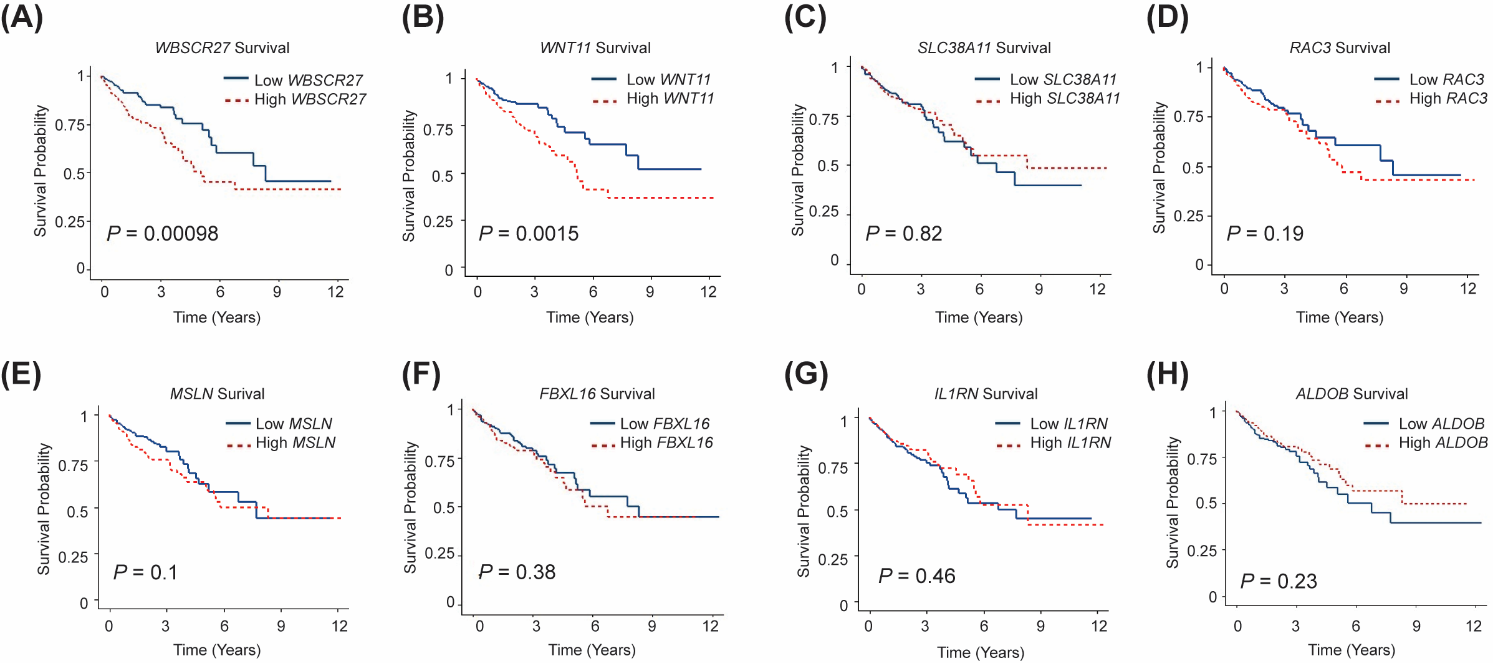
**

**Supplementary Figure 2.** Kaplan-Meier curves comparing EOCRC genes and overall survival in TCGA COAD tumors. **(A**-**F)** Kaplan-Meier curves for each of the eight differentially expressed EOCRC genes and their association with overall survival in TCGA COAD tumors. *P*-values were calculated with the Log-rank test (survival package in R).


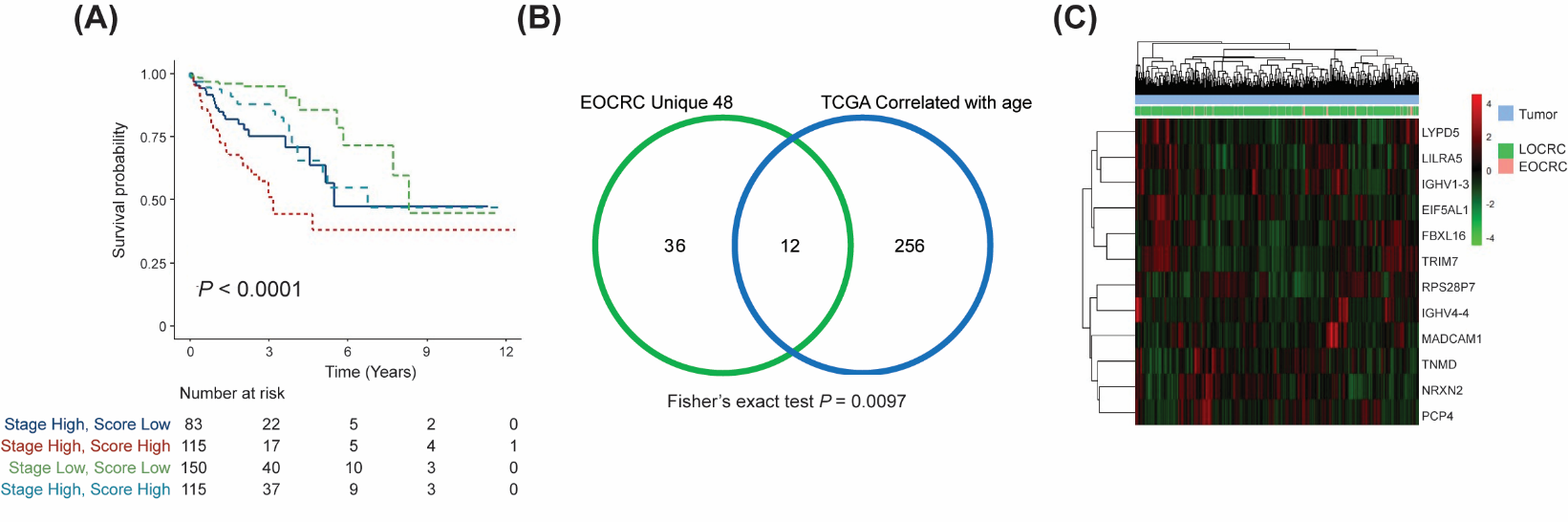


**Supplementary Figure 3.** The TCGA COAD dataset supports age-related genes identified in this study. (**A**) Kaplan-Meier curve from TCGA COAD tumors stratified by tumor stage and the score derived from the eight-gene signature. **(B)** Overlap of genes specific to EOCRC in our dataset and genes correlating with age in the TCGA dataset. **(C)** Heatmap of the 12 genes unique to EOCRC in our dataset and correlating with age in TCGA COAD. Heatmap colors represent the normalized expression of TCGA COAD tumors labeled by LOCRC (green) or EOCRC (coral).


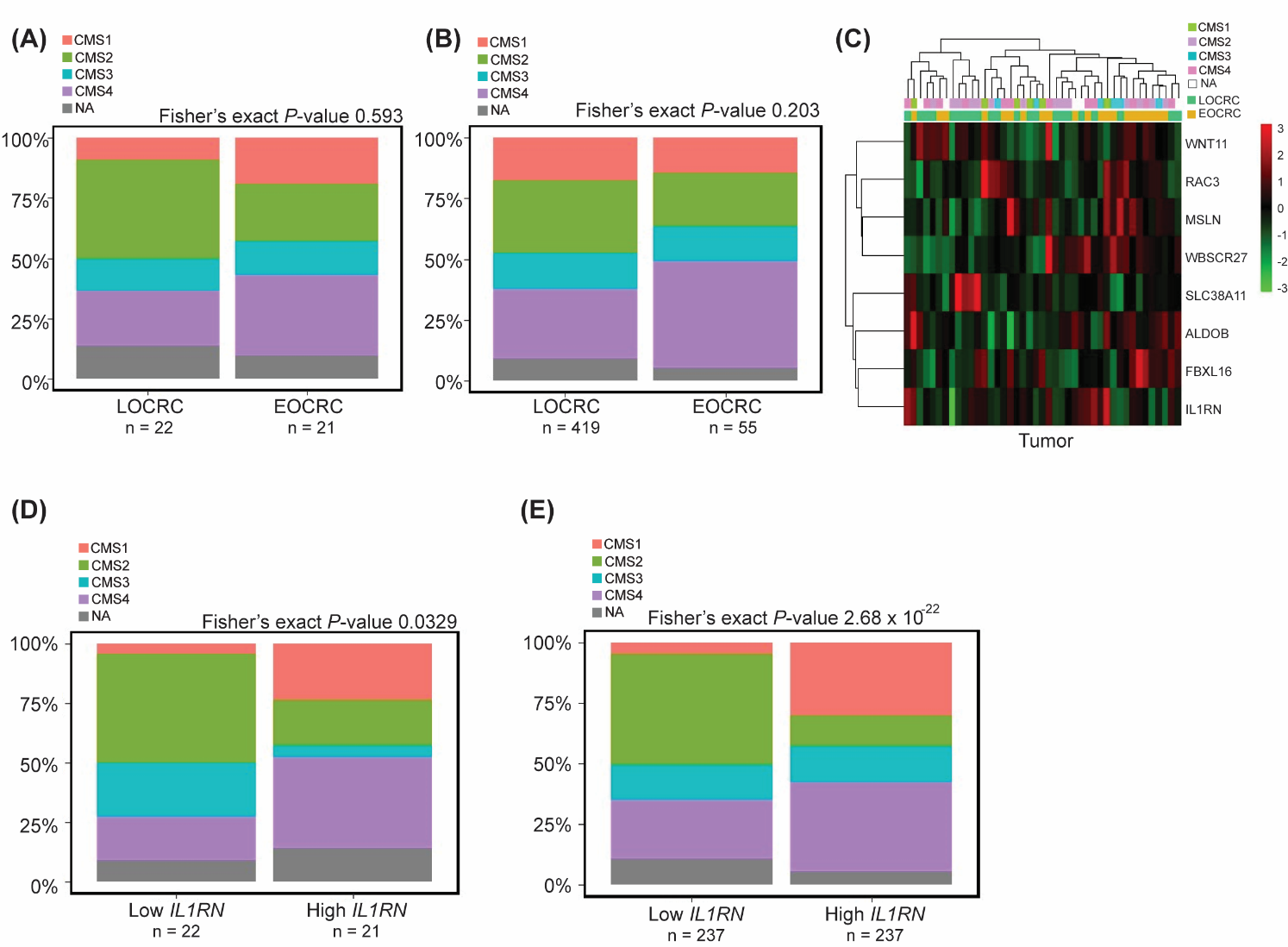


**Supplementary Figure 4.** Predicted CMS do not distinguish EOCRCs and LOCRCs. **(A-B)** Bar plots showing the percentage of CMS1-4 (or NA) values obtained from CMSCaller package in R for our data **(A)** or TCGA COAD data **(B)**. **(C)** Relative expression of eight-gene signature in our EOCRC and LOCRC tumor samples, colored by age group and by CMS, plotted using hierarchical clustering. **(D-E)** CMS for tumor samples from our data **(D)** or TCGA COAD **(E)** separated into high and low groups based on median *IL1RN* expression. Fisher’s exact test was used to compare differences in CMS between EOCRC and LOCRC and between high and low *IL1RN* tumors.

| **Table S1.** Genes significantly differentially expressed in EOCRC. | | | | | | |  |
| --- | --- | --- | --- | --- | --- | --- | --- |
| **Gene** | **EOCRC baseMean** | **EOCRC LFC** | **EOCRC *P*-adj** | **LOCRC baseMean** | **LOCRC LFC** | **LOCRC *P*-adj** | **LFC Difference** |
| TNMD | 53.064 | 1.341 | 0.011 | 71.832 | 0.518 | 0.361 | 0.822 |
| ARHGAP6 | 108.221 | -1.173 | 0.007 | 239.809 | -0.415 | 0.358 | -0.758 |
| WNT11 | 159.267 | 1.801 | 0.001 | 197.291 | 0.527 | 0.271 | 1.274 |
| MADCAM1 | 64.201 | -1.215 | 0.018 | 56.038 | -0.108 | 0.847 | -1.107 |
| MSLN | 763.441 | 2.336 | 0.000 | 545.214 | 0.491 | 0.303 | 1.845 |
| TFPI2 | 71.988 | 1.227 | 0.023 | 381.973 | 0.692 | 0.356 | 0.535 |
| NPTX2 | 193.190 | 1.846 | 0.001 | 111.581 | 0.618 | 0.339 | 1.228 |
| PTGDS | 77.180 | -1.512 | 0.006 | 80.228 | -0.422 | 0.331 | -1.090 |
| NRXN2 | 104.933 | -1.327 | 0.006 | 198.185 | -0.203 | 0.720 | -1.125 |
| POU2AF1 | 471.442 | -1.378 | 0.005 | 551.288 | -0.355 | 0.510 | -1.023 |
| CCR7 | 178.625 | -1.038 | 0.045 | 103.818 | -0.440 | 0.294 | -0.598 |
| HSPA2 | 539.592 | -1.879 | 0.000 | 1489.187 | -0.381 | 0.482 | -1.498 |
| FBXL16 | 74.271 | 1.191 | 0.002 | 55.014 | 0.109 | 0.791 | 1.082 |
| STEAP4 | 110.370 | -1.506 | 0.004 | 193.202 | -0.441 | 0.356 | -1.066 |
| HOXD9 | 100.192 | -1.200 | 0.024 | 132.598 | -0.240 | 0.664 | -0.960 |
| AVIL | 109.735 | -1.003 | 0.000 | 248.383 | -0.373 | 0.274 | -0.630 |
| IL1RN | 573.917 | 1.348 | 0.015 | 525.447 | 0.476 | 0.234 | 0.872 |
| ALDOB | 2223.271 | 2.601 | 0.000 | 1797.235 | 0.942 | 0.151 | 1.660 |
| DUOX2 | 3155.243 | 1.509 | 0.002 | 6251.065 | 0.386 | 0.465 | 1.123 |
| TRIM7 | 90.442 | 1.359 | 0.009 | 88.875 | 0.175 | 0.772 | 1.183 |
| STC1 | 178.058 | 1.734 | 0.001 | 315.649 | 0.555 | 0.328 | 1.179 |
| LYPD5 | 59.104 | 1.867 | 0.000 | 76.542 | 0.557 | 0.207 | 1.310 |
| WBSCR27 | 165.775 | 1.150 | 0.018 | 123.062 | 0.343 | 0.442 | 0.808 |
| SLC38A11 | 52.291 | -1.226 | 0.002 | 159.315 | 0.301 | 0.577 | -1.527 |
| CLIC3 | 75.933 | 1.056 | 0.025 | 57.594 | -0.309 | 0.407 | 1.365 |
| RAC3 | 87.243 | 1.052 | 0.001 | 84.992 | 0.109 | 0.737 | 0.942 |
| FIBIN | 90.936 | 1.058 | 0.013 | 208.488 | 0.544 | 0.262 | 0.515 |
| LCN15 | 127.469 | -2.833 | 0.000 | 333.932 | 0.873 | 0.365 | -3.706 |
| TMEM45A | 170.282 | 1.192 | 0.013 | 184.762 | -0.077 | 0.832 | 1.269 |
| PCP4 | 152.893 | 1.814 | 0.032 | 230.895 | -1.732 | 0.019 | 3.546 |
| PRKD1 | 54.392 | -1.028 | 0.003 | 108.366 | -0.252 | 0.512 | -0.776 |
| IFITM2 | 2643.349 | 1.190 | 0.003 | 3389.881 | 0.405 | 0.374 | 0.785 |
| C16orf54 | 106.867 | -1.369 | 0.000 | 172.702 | -0.207 | 0.733 | -1.163 |
| LILRA5 | 73.734 | 1.092 | 0.034 | 83.791 | 0.577 | 0.201 | 0.515 |
| L1CAM | 116.625 | -1.243 | 0.016 | 310.987 | -0.004 | 0.996 | -1.238 |
| IGLV5-45 | 260.592 | -1.390 | 0.016 | 407.262 | -0.541 | 0.414 | -0.849 |
| IGLV2-18 | 323.825 | -1.319 | 0.034 | 876.393 | -0.386 | 0.564 | -0.933 |
| IGHV1-3 | 882.319 | -1.363 | 0.039 | 971.959 | 0.048 | 0.951 | -1.411 |
| IGHV3-13 | 119.681 | -1.608 | 0.006 | 382.981 | 0.402 | 0.587 | -2.010 |
| IGHV4-39 | 1608.259 | -1.310 | 0.046 | 2508.769 | -0.130 | 0.849 | -1.180 |
| IGHV3-73 | 194.850 | -1.099 | 0.042 | 399.903 | -0.259 | 0.690 | -0.840 |
| MEG3 | 202.360 | -1.068 | 0.010 | 384.040 | -0.253 | 0.549 | -0.815 |
| IGHV3-74 | 882.384 | -1.315 | 0.023 | 1785.759 | -0.386 | 0.517 | -0.929 |
| RPS28P7 | 1647.814 | 1.322 | 0.029 | 811.457 | 0.495 | 0.207 | 0.827 |
| IGKV1-12 | 665.375 | -1.247 | 0.015 | 1371.295 | -0.634 | 0.277 | -0.613 |
| EIF5AL1 | 62.205 | 1.407 | 0.005 | 122.084 | 0.424 | 0.375 | 0.983 |
| IGHV2-70 | 152.202 | -1.407 | 0.033 | 180.176 | 0.359 | 0.665 | -1.766 |
| IGHV4-4 | 516.062 | -1.652 | 0.006 | 603.695 | -0.327 | 0.604 | -1.325 |

BaseMean gives the mean counts for the gene in each cohort. LFC is the log2 fold change in tumors compared to normal samples. *P*-adj is the adjusted p-value. LFC Dif is the difference in LFC in EOCRC versus LOCRC. Values rounded to 3 decimal points.

| **Table S2:** Eight-gene signature differentially expressed in EOCRC. | | | | | |
| --- | --- | --- | --- | --- | --- |
| **Gene** | **EOCRC LFC** | **EOCRC  *P*-adj** | **LOCRC LFC** | **LOCRC**  ***P*-adj** | **Description** |
| **WNT11** | 1.801 | 0.001 | 0.527 | 0.271 | Non-canonical Wnt ligand that is upregulated in some CRCs and promotes invasion and migration(1, 2). Wnt11 has also been shown to reduce canonical Wnt/β-catenin signaling(3, 4). |
| **MSLN** | 2.336 | 0.000 | 0.491 | 0.303 | Encodes the cell surface protein mesothelin, which is increased in CRC. In CRC, MSLN-staining positive CRCs were slightly younger (p = 0.093), and MSLN-high patients had worse overall survival(5). MSLN is a target of CAR-T therapy and is currently undergoing clinical trials for its utility in solid tumors including CRC(6). |
| **FBXL16** | 1.191 | 0.002 | 0.109 | 0.791 | E3 ubiquitin ligase that may stabilize c-MYC by blocking its interaction with FBW7(7). FBXL16 may promote lung cancer by stabilizing IRS1 and upregulating PI3K/AKT signaling(8). |
| **IL1RN** | 1.348 | 0.015 | 0.476 | 0.234 | Agonist that binds interleukin IL-1 surface receptor (IL1R1), repressing its activity. In CRC, IL1RN is upregulated and positively regulates invasion and metastasis(9) and is positively correlated with immune infiltration(10). |
| **WBSCR27** | 1.150 | 0.018 | 0.343 | 0.442 | Methyltransferase-like protein that is upregulated in CRC tumors versus normal samples and associated with poor prognosis and low immune invasion(11). The exact function and substrate of this protein remain elusive(12). |
| **SLC38A11** | -1.226 | 0.002 | 0.301 | 0.577 | Relatively uncharacterized amino acid transporter with suggested roles in metabolism(13). |
| **RAC3** | 1.052 | 0.001 | 0.109 | 0.737 | Transcriptional coactivator of NF-κB(14) that is increased many cancers, including CRC. *RAC3* is increased in CRC tumors and metastases, and its expression is associated with CRC stemness and markers of epithelial-mesenchymal transition(15). RAC3 decreases with aging in rats and may inhibit senescence(16). |
| **ALDOB** | 2.601 | 0.000 | 0.942 | 0.151 | Encodes Aldolase B gene, induces lactate secretion and high expression is correlated with poor prognosis, cell growth, and chemotherapy resistance in CRC(17). |

LFC is log2 fold change in expression in tumors versus normal samples. *P*-adj represents the adjusted p-value from DESeq2 output, and description includes known roles of each gene in cancer and beyond. The Description column contains a summary of the known roles of each gene relating to CRC. Values rounded to 3 decimal places.

| **Table S3.** Prognostic indicators in TCGA COAD tumors. | | | | |
| --- | --- | --- | --- | --- |
| **Clinical Characteristic** | **HR** | **95% CI** | **Z-score** | ***P*-value** |
| Gender Male | 1.41 | (.30, 1.69) | -0.78 | 0.44 |
| Lymphatic invasion present | 0.33 | (1.05, 8.85) | 2.04 | **0.04** |
| Mucinous Adenocarcinoma | 0.41 | (0.75, 7.90) | 1.48 | 0.14 |
| Microsatellite instable- low | 0.49 | (0.50, 8.45) | 0.99 | 0.32 |
| Microsatellite stable | 1.46 | (0.24, 1.96) | -0.71 | 0.48 |
| Stage low (I/II) | 4.20 | (0.03, 1.93) | -1.34 | 0.18 |
| Tumor stage low (I/II) | 4.51 | (0.03, 1.78) | -1.42 | 0.16 |
| Metastasis present | 0.70 | (0.47, 4.35) | 0.62 | 0.53 |
| Node positive | 2.92 | (0.05, 2.22) | -1.12 | 0.26 |
| Age under 50 | 1.18 | (0.18, 4.09) | -0.20 | 0.84 |
| 8GeneScore High | 0.31 | (1.08, 9.77) | 2.10 | **0.04** |

HR is hazard ratio. CI is confidence interval. Clinical characteristics were separated into binary measurements when possible and Cox proportional hazard regression was used to examine relationship with survival from 462 patients with data available from UCSC Xenabrowser. 8GeneScore represents the score generated by the eight gene signature specifically differentially expressed in EOCRC.

| **Table S4.** Eight-gene signature association with CMS.   \| **Gene** \| **Our CMS** \| **TCGA CMS** \| \| --- \| --- \| --- \| \| WBSCR27 \| 0.7238 \| 0.6910 \| \| IL1RN \| 0.0329 \| 4.509E-22 \| \| RAC3 \| 0.4883 \| 3.073E-10 \| \| FBXL16 \| 0.4883 \| 4.605E-15 \| \| WNT11 \| 0.6757 \| 3.821E-09 \| \| SLC38A11 \| 0.1142 \| 1.596E-07 \| \| ALDOB \| 0.1382 \| 0.1182 \| \| MSLN \| 0.9346 \| 0.0938 \|   Data represents *P*-values from Fisher’s exact tests for the relationship between high or low tumor expression of each gene (separated by median) and the predicted consensus molecular subtypes for our data (n = 43 tumors) and TCGA COAD data (n = 474 tumors). |
| --- | --- | --- | --- | --- | --- | --- | --- | --- | --- | --- | --- | --- | --- | --- | --- | --- | --- | --- | --- | --- | --- | --- | --- | --- | --- | --- | --- |

| **Table S5:** Significant KEGG terms in EOCRC and LOCRC | | | | | | | | | | |
| --- | --- | --- | --- | --- | --- | --- | --- | --- | --- | --- |
| **KEGG Term** | **Size** | **EO-CRC ES** | **EO-CRC NES** | **EO-CRC NOM *P*-val** | **EO-CRC FDR q-val** | **LO-CRC ES** | **LO-CRC NES** | **LO-CRC NOM *P* -val** | **LO-CRC FDR q-val** | **Sig** |
| KEGG_DNA_REPLICATION | 36 | 0.81 | 1.6 | 0 | 0.096 | 0.82 | 1.44 | 0.006 | 0.191 | Both |
| KEGG_MISMATCH_REPAIR | 23 | 0.78 | 1.6 | 0.002 | 0.139 | 0.75 | 1.39 | 0.046 | 0.227 | Both |
| KEGG_NUCLEOTIDE_EXCISION_REPAIR | 44 | 0.64 | 1.59 | 0.004 | 0.084 | 0.67 | 1.47 | 0.026 | 0.314 | Both |
| KEGG_HOMOLOGOUS_RECOMBINATION | 28 | 0.74 | 1.52 | 0.006 | 0.09 | 0.76 | 1.36 | 0.036 | 0.279 | Both |
| KEGG_BASE_EXCISION_REPAIR | 35 | 0.65 | 1.56 | 0.012 | 0.083 | 0.69 | 1.47 | 0.013 | 0.243 | Both |
| KEGG_AMINOACYL_TRNA_BIOSYNTHESIS | 41 | 0.69 | 1.64 | 0.015 | 0.171 | 0.71 | 1.46 | 0.072 | 0.21 | EO |
| KEGG_CELL_CYCLE | 125 | 0.61 | 1.53 | 0.017 | 0.094 | 0.62 | 1.42 | 0.05 | 0.201 | EO |
| KEGG_RNA_DEGRADATION | 59 | 0.58 | 1.55 | 0.02 | 0.079 | 0.66 | 1.63 | 0.006 | 0.091 | Both |
| KEGG_RNA_POLYMERASE | 29 | 0.69 | 1.5 | 0.021 | 0.091 | 0.67 | 1.45 | 0.044 | 0.186 | Both |
| KEGG_ONE_CARBON_POOL_BY_FOLATE | 17 | 0.68 | 1.51 | 0.026 | 0.093 | 0.6 | 1.29 | 0.177 | 0.26 | EO |
| KEGG_SPLICEOSOME | 127 | 0.53 | 1.58 | 0.033 | 0.073 | 0.6 | 1.56 | 0.019 | 0.145 | Both |
| KEGG_OLFACTORY_TRANSDUCTION | 388 | -0.4 | -1.65 | 0 | 0.516 | -0.42 | -1.72 | 0 | 0.095 | Both |
| KEGG_INOSITOL_PHOSPHATE_METABOLISM | 54 | -0.64 | -1.57 | 0.002 | 0.621 | -0.61 | -1.45 | 0.05 | 0.785 | EO |
| KEGG_PROXIMAL_TUBULE_BICARBONATE_RECLAMATION | 23 | -0.74 | -1.55 | 0.006 | 0.535 | -0.69 | -1.32 | 0.085 | 0.455 | EO |
| KEGG_INSULIN_SIGNALING_PATHWAY | 137 | -0.53 | -1.6 | 0.008 | 0.607 | -0.5 | -1.39 | 0.056 | 0.641 | EO |
| KEGG_NEUROTROPHIN_SIGNALING_PATHWAY | 126 | -0.54 | -1.55 | 0.008 | 0.439 | -0.52 | -1.41 | 0.079 | 0.827 | EO |
| KEGG_ALDOSTERONE_REGULATED_SODIUM_REABSORPTION | 41 | -0.65 | -1.53 | 0.012 | 0.468 | -0.7 | -1.37 | 0.042 | 0.6 | Both |
| KEGG_LONG_TERM_DEPRESSION | 70 | -0.57 | -1.48 | 0.013 | 0.466 | -0.58 | -1.33 | 0.077 | 0.464 | EO |
| KEGG_TASTE_TRANSDUCTION | 51 | -0.64 | -1.51 | 0.019 | 0.495 | -0.49 | -1.17 | 0.267 | 0.441 | EO |
| KEGG_PHOSPHATIDYLINOSITOL_SIGNALING_SYSTEM | 76 | -0.56 | -1.48 | 0.019 | 0.488 | -0.54 | -1.38 | 0.073 | 0.613 | EO |
| KEGG_STARCH_AND_SUCROSE_METABOLISM | 52 | -0.75 | -1.47 | 0.023 | 0.465 | -0.73 | -1.43 | 0.021 | 0.888 | Both |
| KEGG_GLYCEROPHOSPHOLIPID_METABOLISM | 77 | -0.47 | -1.4 | 0.026 | 0.47 | -0.53 | -1.39 | 0.042 | 0.667 | Both |
| KEGG_ADIPOCYTOKINE_SIGNALING_PATHWAY | 67 | -0.56 | -1.48 | 0.029 | 0.542 | -0.56 | -1.33 | 0.104 | 0.477 | EO |
| KEGG_GNRH_SIGNALING_PATHWAY | 101 | -0.5 | -1.43 | 0.03 | 0.558 | -0.52 | -1.38 | 0.028 | 0.652 | Both |
| KEGG_CALCIUM_SIGNALING_PATHWAY | 178 | -0.59 | -1.42 | 0.031 | 0.48 | -0.6 | -1.33 | 0.086 | 0.49 | EO |
| KEGG_VASOPRESSIN_REGULATED_WATER_REABSORPTION | 44 | -0.53 | -1.48 | 0.037 | 0.582 | -0.57 | -1.49 | 0.027 | 0.708 | Both |
| KEGG_ERBB_SIGNALING_PATHWAY | 87 | -0.49 | -1.42 | 0.042 | 0.467 | -0.5 | -1.36 | 0.074 | 0.566 | EO |
| KEGG_ENDOCYTOSIS | 181 | -0.46 | -1.43 | 0.047 | 0.527 | -0.51 | -1.42 | 0.058 | 0.825 | EO |
| KEGG_REGULATION_OF_ACTIN_CYTOSKELETON | 213 | -0.47 | -1.38 | 0.047 | 0.429 | -0.51 | -1.32 | 0.105 | 0.446 | EO |
| KEGG_RENAL_CELL_CARCINOMA | 70 | -0.5 | -1.44 | 0.049 | 0.53 | -0.53 | -1.36 | 0.102 | 0.546 | EO |
| KEGG_NICOTINATE_AND_NICOTINAMIDE_METABOLISM | 24 | -0.54 | -1.3 | 0.11 | 0.4 | -0.67 | -1.53 | 0.006 | 0.846 | LO |
| KEGG_PANTOTHENATE_AND_COA_BIOSYNTHESIS | 16 | -0.6 | -1.25 | 0.197 | 0.428 | -0.77 | -1.47 | 0.016 | 0.784 | LO |
| KEGG_LYSOSOME | 121 | -0.47 | -1.4 | 0.099 | 0.499 | -0.57 | -1.51 | 0.021 | 0.774 | LO |
| KEGG_SPHINGOLIPID_METABOLISM | 39 | -0.55 | -1.31 | 0.153 | 0.402 | -0.6 | -1.41 | 0.046 | 0.77 | LO |

Size gives the number of genes in KEGG set. ES is enrichment score, NES is normalized ES. NOM *P*-val is nominal *P*-value, FDR q-value is false discovery rate. Significance column represents whether the term is significant in EOCRC (EO), LOCRC (LO), or both.

| **Table S6.** Significantly differentially spliced events in EOCRC. | | | | |
| --- | --- | --- | --- | --- |
| **Gene** | **Location** | **EO dPSI** | **EOCRC FDR** | **Type** |
| PDGFA | chr7 - 500430 500499 | -0.130 | 1.07E-02 | SE |
| DVL1 | chr1 - 1339286 1339439 | 0.113 | 1.75E-03 | A5SS |
| MFSD12 | chr19 - 3542775 3542977 | -0.193 | 1.63E-02 | SE |
| RAC1 | chr7 + 6398661 6398718 | 0.116 | 0.00E+00 | SE |
| SBF2-AS1 | chr11 + 9780375 9780575 | -0.126 | 1.75E-02 | SE |
| KLRK1-AS1 | chr12 + 10373040 10373137 | -0.229 | 9.54E-05 | MXE |
| DNM2 | chr19 + 10808568 10808580 | 0.265 | 1.76E-11 | SE |
| MYH11 | chr16 - 15708802 15708841 | -0.352 | 0.00E+00 | SE |
| PEX26 | chr22 + 18085111 18085258 | -0.104 | 1.61E-04 | SE |
| HR | chr8 - 22116874 22117039 | -0.138 | 4.91E-02 | SE |
| SLC39A14 | chr8 + 22412036 22412206 | 0.256 | 5.30E-09 | SE |
| CYFIP1 | chr15 - 22916881 22917029 | -0.191 | 4.36E-04 | SE |
| HOTAIRM1 | chr7 + 27099097 27099366 | 0.178 | 8.34E-03 | SE |
| TJP1 | chr15 - 29719776 29720016 | -0.158 | 2.64E-05 | SE |
| MAP3K8 | chr10 + 30437175 30437406 | -0.110 | 1.96E-05 | SE |
| GK | chrX + 30727465 30727552 | 0.177 | 7.94E-12 | SE |
| SMTN | chr22 + 31100884 31100953 | 0.291 | 0.00E+00 | SE |
| SMTN | chr22 + 31100884 31101049 | 0.180 | 0.00E+00 | SE |
| HLA-DMB | chr6 - 32935341 32935377 | -0.146 | 1.26E-08 | SE |
| PARD3 | chr10 - 34337274 34337426 | 0.142 | 2.99E-08 | A5SS |
| PARD3 | chr10 - 34337277 34337426 | 0.161 | 3.46E-06 | A5SS |
| AP2B1 | chr17 + 35670856 35670898 | -0.163 | 1.17E-06 | SE |
| SNHG11 | chr20 + 38447929 38448093 | -0.113 | 2.78E-06 | SE |
| DMPK | chr19 - 45770970 45771396 | 0.309 | 3.86E-06 | SE |
| MADD | chr11 + 47326737 47326807 | -0.101 | 1.61E-05 | SE |
| PLEKHA4 | chr19 - 48839204 48839263 | -0.106 | 2.91E-02 | SE |
| ECHDC2 | chr1 - 52906518 52906611 | -0.241 | 3.31E-09 | SE |
| GCOM1 | chr15 + 57674967 57675068 | -0.282 | 8.02E-13 | MXE |
| MSI2 | chr17 + 57676986 57677059 | 0.153 | 4.34E-02 | SE |
| FLNB | chr3 + 58131926 58132019 | -0.192 | 2.94E-05 | SE |
| TPM1 | chr15 + 63043705 63044152 | 0.259 | 1.75E-08 | A5SS |
| TPM1 | chr15 + 63043705 63043831 | -0.255 | 1.28E-07 | SE |
| TPM1 | chr15 + 63043705 63043831 | -0.253 | 6.88E-08 | MXE |
| MACROD1 | chr11 - 63998837 63998872 | -0.133 | 6.90E-06 | SE |
| LAS1L | chrX - 65524563 65524614 | -0.112 | 2.99E-10 | SE |
| C8orf44 | chr8 + 66677641 66677954 | -0.113 | 2.72E-09 | SE |
| ACTN1 | chr14 - 68878457 68878520 | -0.191 | 1.37E-07 | SE |
| ACTN1 | chr14 - 68878457 68878523 | 0.215 | 8.91E-10 | MXE |
| ACTN1 | chr14 - 68878457 68878520 | 0.174 | 1.53E-08 | MXE |
| ACTN1 | chr14 - 68878457 68878523 | -0.189 | 2.95E-07 | SE |
| ACTN1 | chr14 - 68878457 68879069 | -0.108 | 2.77E-07 | A5SS |
| TIA1 | chr2 - 70225327 70225393 | -0.127 | 1.47E-02 | SE |
| CTTN | chr11 + 70421469 70421580 | 0.277 | 0.00E+00 | SE |
| VCL | chr10 + 74111908 74112112 | -0.144 | 1.91E-04 | SE |
| USO1 | chr4 + 75795335 75795356 | -0.163 | 1.57E-02 | SE |
| ACOX1 | chr17 - 75960214 75960375 | -0.102 | 2.85E-03 | MXE |
| RPS24 | chr10 + 78040203 78040225 | -0.214 | 1.22E-12 | SE |
| STARD5 | chr15 - 81323801 81324141 | 0.137 | 1.75E-03 | A5SS |
| SEC31A | chr4 - 82830936 82830975 | -0.201 | 7.15E-05 | SE |
| HIKESHI | chr11 + 86345093 86345198 | -0.177 | 0.00E+00 | SE |
| MBNL2 | chr13 + 97366458 97366553 | 0.164 | 6.18E-03 | SE |
| HPS1 | chr10 - 98433939 98434091 | 0.106 | 2.00E-03 | A5SS |
| PAM | chr5 + 102974115 102974436 | -0.208 | 1.00E-08 | SE |
| FHL2 | chr2 - 105396646 105396697 | -0.106 | 4.59E-03 | SE |
| FHL2 | chr2 - 105396646 105396697 | -0.118 | 3.62E-04 | SE |
| TPD52L1 | chr6 + 125253716 125253755 | -0.139 | 1.95E-04 | SE |
| CALD1 | chr7 + 134935687 134935765 | -0.307 | 5.29E-12 | SE |
| MACROH2A1 | chr5 - 135350822 135350913 | 0.205 | 0.00E+00 | MXE |
| MACROH2A1 | chr5 - 135350822 135350913 | -0.187 | 0.00E+00 | SE |
| REPS1 | chr6 - 138926400 138926481 | 0.235 | 1.83E-11 | SE |
| REPS1 | chr6 - 138926400 138930098 | 0.124 | 3.01E-11 | A5SS |
| REPS1 | chr6 - 138926400 138926478 | 0.257 | 1.96E-09 | SE |
| PTK2 | chr8 - 140669726 140669735 | -0.154 | 2.19E-11 | SE |
| ARFIP1 | chr4 + 152870752 152870848 | -0.135 | 1.45E-07 | SE |
| ZBTB7B | chr1 + 155014014 155014093 | -0.122 | 1.95E-04 | SE |
| ADAM15 | chr1 + 155061903 155062117 | -0.171 | 9.83E-09 | SE |
| ADAM15 | chr1 + 155061903 155062117 | -0.142 | 2.57E-02 | SE |
| ADAM15 | chr1 + 155061903 155061975 | -0.159 | 1.03E-03 | SE |
| ADAM15 | chr1 + 155061903 155061975 | -0.103 | 0.00E+00 | SE |
| CLK2 | chr1 - 155268707 155268795 | 0.101 | 4.00E-02 | SE |
| PDLIM7 | chr5 - 177491403 177491420 | 0.324 | 0.00E+00 | MXE |
| PDLIM7 | chr5 - 177491403 177491420 | 0.267 | 0.00E+00 | MXE |
| PDLIM7 | chr5 - 177491806 177491976 | 0.380 | 0.00E+00 | SE |
| PDLIM7 | chr5 - 177491806 177491925 | 0.304 | 0.00E+00 | SE |
| PDLIM7 | chr5 - 177491806 177491995 | 0.385 | 0.00E+00 | SE |
| CRYZL2P | chr1 - 178022439 178022668 | -0.121 | 5.63E-06 | SE |
| CRYZL2P | chr1 - 178022439 178022668 | 0.116 | 1.47E-03 | MXE |
| OPA1 | chr3 + 193626091 193626202 | -0.101 | 2.87E-03 | MXE |
| OPA1 | chr3 + 193626091 193626202 | -0.168 | 7.55E-08 | SE |
| CLK1 | chr2 - 200860124 200860215 | 0.170 | 0.00E+00 | SE |
| CD46 | chr1 + 207767778 207767823 | -0.102 | 2.00E-10 | SE |
| CD46 | chr1 + 207767778 207767823 | -0.110 | 1.06E-06 | MXE |

FDR is false discovery rate from rMATS analysis. Splice types: skipped exon (SE), mutually exclusive exons (MXE), and alternative 5’ splice site (A5SS). Location represents the chromosome, strand, and beginning and end of an exon involved in the splice event as defined by rMATS in human genome build hg38. For A5SS, coordinates represent the long exon start and long exon end, respectively. For SE, coordinates represent the start and end of the skipped exon. For MXE, coordinates represent the first exon start and first exon end. dPSI represents the change in percent spliced in between tumor and normal samples from rMATS analysis for early-onset CRCs.

**
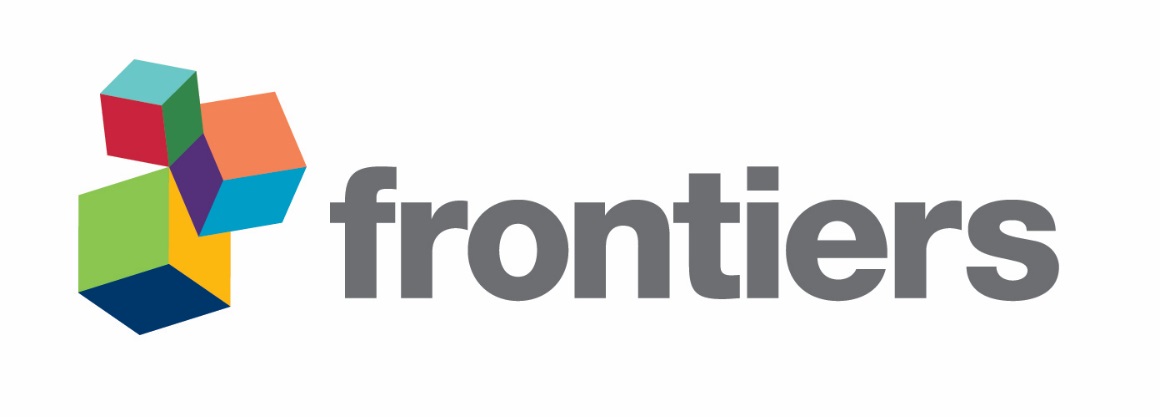
**

**Supplemental References**

1. Gorroño-Etxebarria I, Aguirre U, Sanchez S, González N, Escobar A, Zabalza I, et al. Wnt-11 as a Potential Prognostic Biomarker and Therapeutic Target in Colorectal Cancer. Cancers (Basel). 2019;11(7).

2. Nishioka M, Ueno K, Hazama S, Okada T, Sakai K, Suehiro Y, et al. Possible involvement of Wnt11 in colorectal cancer progression. Mol Carcinog. 2013;52(3):207-17.

3. Railo A, Nagy, II, Kilpeläinen P, Vainio S. Wnt-11 signaling leads to down-regulation of the Wnt/beta-catenin, JNK/AP-1 and NF-kappaB pathways and promotes viability in the CHO-K1 cells. Exp Cell Res. 2008;314(13):2389-99.

4. Maye P, Zheng J, Li L, Wu D. Multiple mechanisms for Wnt11-mediated repression of the canonical Wnt signaling pathway. J Biol Chem. 2004;279(23):24659-65.

5. Inoue S, Tsunoda T, Riku M, Ito H, Inoko A, Murakami H, et al. Diffuse mesothelin expression leads to worse prognosis through enhanced cellular proliferation in colorectal cancer. Oncol Lett. 2020;19(3):1741-50.

6. Klampatsa A, Dimou V, Albelda SM. Mesothelin-targeted CAR-T cell therapy for solid tumors. Expert Opin Biol Ther. 2021;21(4):473-86.

7. Morel M, Shah KN, Long W. The F-box protein FBXL16 up-regulates the stability of C-MYC oncoprotein by antagonizing the activity of the F-box protein FBW7. J Biol Chem. 2020;295(23):7970-80.

8. Morel M, Long W. FBXL16 promotes cell growth and drug resistance in lung adenocarcinomas with KRAS mutation by stabilizing IRS1 and upregulating IRS1/AKT signaling. Mol Oncol. 2023.

9. Chen Y, Yang Z, Deng B, Wu D, Quan Y, Min Z. Interleukin 1β/1RA axis in colorectal cancer regulates tumor invasion, proliferation and apoptosis via autophagy. Oncol Rep. 2020;43(3):908-18.

10. Wang Q, Huang X, Zhou S, Ding Y, Wang H, Jiang W, et al. IL1RN and PRRX1 as a Prognostic Biomarker Correlated with Immune Infiltrates in Colorectal Cancer: Evidence from Bioinformatic Analysis. Int J Genomics. 2022;2022:2723264.

11. Wang K, Zhang J, Deng M, Ju Y, Ouyang M. [METTL27 is a prognostic biomarker of colon cancer and associated with immune invasion]. Nan Fang Yi Ke Da Xue Xue Bao. 2022;42(4):486-97.

12. Mariasina SS, Chang CF, Navalayeu TL, Chugunova AA, Efimov SV, Zgoda VG, et al. Williams-Beuren Syndrome Related Methyltransferase WBSCR27: From Structure to Possible Function. Front Mol Biosci. 2022;9:865743.

13. Aggarwal T, Patil S, Ceder M, Hayder M, Fredriksson R. Knockdown of SLC38 Transporter Ortholog - CG13743 Reveals a Metabolic Relevance in Drosophila. Front Physiol. 2019;10:1592.

14. Werbajh S, Nojek I, Lanz R, Costas MA. RAC-3 is a NF-kappa B coactivator. FEBS Lett. 2000;485(2-3):195-9.

15. Panelo LC, Machado MS, Rubio MF, Jaworski F, Alvarado CV, Paz LA, et al. High RAC3 expression levels are required for induction and maintaining of cancer cell stemness. Oncotarget. 2018;9(5):5848-60.

16. Fernández Larrosa PN, Ruíz Grecco M, Mengual Gómez D, Alvarado CV, Panelo LC, Rubio MF, et al. RAC3 more than a nuclear receptor coactivator: a key inhibitor of senescence that is downregulated in aging. Cell Death Dis. 2015;6(10):e1902.

17. Chu YD, Cheng LC, Lim SN, Lai MW, Yeh CT, Lin WR. Aldolase B-driven lactagenesis and CEACAM6 activation promote cell renewal and chemoresistance in colorectal cancer through the Warburg effect. Cell Death Dis. 2023;14(10):660.
